# Intermediate Care in Italy: Addressing the Challenges and Opportunities for Person-Tailored Care

**DOI:** 10.3390/geriatrics8030059

**Published:** 2023-05-30

**Authors:** Virginia Boccardi, Patrizia Mecocci

**Affiliations:** 1 Section of Gerontology and Geriatrics, Department of Medicine and Surgery, University of Perugia, Santa Maria della Misericordia Hospital, Piazzale Gambuli 1, 06132 Perugia, Italy; patrizia.mecocci@unipg.it; 2 Division of Clinical Geriatrics, NVS Department, Karolinska Institutet, 17177 Stockholm, Sweden

**Keywords:** aging, challenge, frailty, geriatrics, healthcare

## Abstract

The concept of intermediate care is gaining increasing recognition in Italy as a critical strategy for improving quality of care and promoting the integration of healthcare services across different settings. This is driven by demographic changes and the growing prevalence of chronic conditions. One of the key challenges in delivering intermediate care in Italy is ensuring that care is person-tailored, which requires a shift towards a more holistic approach that prioritizes individual preferences and values. This requires greater collaboration and communication across different healthcare settings and a coordinated approach to the delivery of care that promotes innovation and the use of technology to support remote monitoring and care delivery. Despite these challenges, intermediate care offers significant opportunities with which to enhance the quality of care, reduce healthcare costs, and promote social cohesion as well as community engagement. Overall, a coordinated and comprehensive approach is required to address the challenges and opportunities associated with intermediate care and to deliver person-tailored care that improves health outcomes as well as sustainability in Italy.

According to the World Health Organization (WHO), the amount of the world’s population aged 60 and over is projected to reach 2.1 billion by 2050, up from 962 million in 2017. This demographic shift is expected to increase age-related chronic conditions, including dementia, cardiovascular disease, and musculoskeletal disorders [1]. Disability rates are also projected to increase because of population aging, with an estimated 15% of people aged 60 and over currently experiencing significant disability [2].

In Italy, the population is also aging rapidly. As stated by the Italian National Institute of Statistics (ISTAT), the proportion of people over 65 increased from 12.4% in 2002 to 22.3% in 2021 [3]. This demographic shift is expected to rise, with projections suggesting that this age group will reach 27.9% by 2050. As in other countries, the aging of the population in Italy is associated with an increase in chronic diseases—including hypertension, osteoarthritis, and diabetes—and disabilities [4,5]. The prevalence of disability in Italy is also high among older adults. A survey conducted by the ISTAT in 2019 showed that around 37% of people over 65 reported some form of disability, with mobility-related ones being the most common [6]. Thus, if there has been a progressive increase in life expectancy, there has also been a significant lengthening of the years lived with multiple comorbidities and disabilities, with a great impact on all aspects of society, including healthcare, social, and economic issues. The progressive aging of the Italian population has led to greater complexity in caring for older persons within healthcare structures. The term “chronicity” has gradually assumed a connotation of “false” or presumed stability of clinical presentation. In fact, there is nothing more clinically unstable than a defined chronicity of varying severity and disability that requires highly personalized therapeutic and care interventions. A chronic illness condition of an older patient is not static but dynamic, in continuous and constant evolution. There are almost three types of patients who require such a plan, and they are defined as followed: (1) chronic critical illness (severe patients from intensive care units); (2) hospital-dependent patients (chronic patients with high instability); and (3) post-hospital syndrome (those who have been heavily affected by the harmful effects of an acute event and hospitalization in departments where there is a scarce geriatric culture).

The hospital, still representing a reference point for health needs, is increasingly crowded and in crisis. As a consequence, difficulties and challenges arise in the discharge of older patients due to complex medical conditions, social factors, and limited resources for ongoing care. In recent decades in Italy, three fundamental phenomena have created a complex situation in the care of non-self-sufficient people at the time of hospital discharge: firstly, changes in healthcare policies—from an approach based on hospitalization with the financing of hospital services by the National Health Service (NHS) based on hospitalization days to a policy that substantially promotes reductions in hospital stays linked to the care of acute illness in a logic of cost containment; secondly, the complexity of a subject who, after the acute phase, is in a condition of high vulnerability, often related to the lack of a family and social support network; and, thirdly, the social changes in the family structure, which are unable to accommodate and care for an older person. Thus, the continuity of care is fundamental.

The term “intermediate care” was introduced in the United Kingdom in response to a request for an improvement in the care of older people, representing a “concept” rather than a service [7]. Beyond the various definitions and interpretations at the national and international levels, intermediate care has been conceived as a mode of intervention through the participation of multiple services and based on multidimensional evaluation. The concept is that an older person who is in difficulty due to an acute event must find adequate answers that, if at home, avoid unnecessary and inappropriate hospitalization and, if already hospitalized, facilitate their discharge by preparing a proper care path. In Italy, the hospital still plays a central role in acute care, while “intermediate care” or “transition care”, whose concepts date to the early 1990s, are intended for those care services that people use after being discharged from the hospital and before returning home. Transition care consists of services focused on a multidimensional approach and the development of an individual care path with the primary objective of maximum functional recovery and a subsequent return home. Thus, the primary purpose of transition care is to allow an individual—after the resolution of the acute clinical problem that required hospitalization—to achieve maximum functional recovery through rehabilitation and maintenance of functions, enabling a return home and avoiding an improper lengthening of a hospital stay. It is clear that within the intermediate care path, several elements play a central and priority role: (1) CGA (comprehensive geriatric assessment) and an individualized as well as personalized path, (2) bidirectional communication between a hospital and territorial services, (3) uniform operational modalities between a hospital and territory, (4) the sharing of patient information, (5) the establishment of an integrated team composed of different professional figures (nurses, physiotherapists, nutritionists, etc.), and (6) ensuring the continuity of care.

In recent years, also “thanks” to the challenges and opportunities brought about by COVID-19, Italy has experienced a revolution in elderly care, intermediate care, and technology, driven by demographic changes and a growing recognition of the importance of person-tailored care. One of the key changes has been the increased availability and use of intermediate care services, including skilled nursing facilities, rehabilitation centers, and home care services. These services provide a range of supports and interventions, from basic assistance daily activities to more specialized medical care and rehabilitation. At the same time, there has been a growing emphasis on using technology to support elderly care and intermediate care. Technology includes developing remote monitoring systems, telemedicine, and other digital tools that allow healthcare providers to monitor patients and communicate with them more effectively; however, there are substantial regional differences in intermediate care in Italy, with variations in the availability and quality of services across different areas of the country. In general, the northern regions have more advanced and better-funded intermediate care systems, with a higher concentration of services. These regions also tend to have a higher proportion of geriatricians and other specialized healthcare professionals, which can improve the quality of care for non-self-sufficient individuals. In contrast, the southern regions tend to have more limited intermediate care resources, with fewer services available. This can be due to a range of factors, including economic disparities, workforce shortages, and limited access to healthcare infrastructure; however, efforts are being made to address these regional disparities and improve the quality as well as availability of intermediate care services across the country. Such efforts include national policies and initiatives with which to promote the integration and coordination of care across different settings, as well as efforts to strengthen workforce capacity and encourage the use of technology. Another critical challenge in delivering intermediate care in Italy is ensuring that services are accessible, affordable, and sustainable. To allow for this, a coordinated approach to care delivery is required, focusing on promoting innovation and using technology to support remote monitoring in addition to care delivery. There is also a need to promote greater efficiency in the use of resources and to address workforce shortages, particularly in rural and remote areas.

A crucial opportunity is the PNNA (Piano Nazionale Non-Autosufficienza e Riabilitazione) [8]. The PNNA is a national plan in Italy that focuses on caring for and rehabilitating non-self-sufficient individuals, including older adults and those with disabilities. The program aims to improve the quality and availability of intermediate care services, including skilled nursing facilities, rehabilitation centers, and other residential care settings. In addition, the PNNA recognizes the importance of providing comprehensive, person-centered care that is tailored to the needs of everyone. The challenge is to address physical, cognitive, and emotional needs, as well as provide support for family members and caregivers.

One of the main goals of the PNNA is to promote the integration and coordination of care across different settings, including hospitals, intermediate care facilities, and home care. The plan includes developing guidelines and protocols for transferring patients between different care settings in addition to promoting the use of technology and telemedicine to support remote monitoring and communication. The PNNA represents an important step towards improving the quality and availability of intermediate care services as well as promoting a more integrated, person-centered approach to care for non-self-sufficient individuals. In addition, a significant investment should be the increasing role of geriatricians in acute care settings, including hospitals and intermediate care services. These specialized professionals are trained to provide comprehensive, person-centered care to older adults, including those with complex medical needs and frailty.

Overall, the revolution in elderly care, intermediate care, and technology in Italy has significantly improved the quality of care for older adults and those with disabilities; however, addressing the challenges and opportunities associated with intermediate care requires a coordinated and comprehensive approach that prioritizes person-centered care, integration across different settings, and technology as well as innovation to enhance care outcomes and sustainability. By promoting person-tailored care, interdisciplinary collaboration, and innovative technologies, Italy is paving the way for a more responsive, inclusive, and sustainable care system for its aging population.
